# The influences of SE infection on layers’ production performance, egg quality and blood biochemical indicators

**DOI:** 10.1186/2049-1891-5-4

**Published:** 2014-01-09

**Authors:** Shijie Fan, Jiangxia Zheng, Zhongyi Duan, Ning Yang, Guiyun Xu

**Affiliations:** 1 Department of Animal Genetics and Breeding, National Engineering Laboratory for Animal Breeding, MOA Key Laboratory of Animal Genetics and Breeding, China Agricultural University, Beijing 100193, China

**Keywords:** Blood biochemical indicators, Chicken, Egg quality, Salmonella enterica serovar Enteritidis

## Abstract

**Background:**

Salmonella enterica serovar Enteritidis (SE), as a major cause of foodborn illness, infects humans mainly through the egg. However, the symptom of laying hens usually is not typical and hard to diagnosis. In the present study, it is studied that the influences of SE infection on layers’ performance, egg quality and blood biochemical indicators. It will help us to improve the strategy to control SE infection in commercial layers. One hundred layers at 20 wk of age were divided into 2 groups, 60 hens for experiment and others for control. The experiment group was fed with the dosage of 10^8^ CFU SE per hen. The specific PCR was used to detect the deposition of SE. On the 8 d after SE infection, 10 hens from the control group and 30 hens from the experimental group were slaughtered to detect the SE colonization. The production performance, egg quality and blood biochemical indices were also analyzed.

**Results:**

The results showed that the colonization rate of SE was highest in caecum contents (55.17%) and lowest in vagina (17.24%). For the eggs the detection rate of SE was highest on the eggshell (80.00%) and lowest in yolk (18.81%). SE infection had no significant influence on production performance and egg qualities (*P* > 0.05). The difference of laying rate between the experimental and control groups was less than 0.30%, and both were approximately equal to 82.00%. The blood analysis showed that the aspartic aminotransferase (AST) and alanine aminotransferase (ALT) of experimental group was significantly higher than those of control group *(P* < 0.05). For experimental and control groups AST values were 236.22 U/l and 211.84 U/l respectively, and ALT values were 32.19 U/l and 24.55 U/l. All of coefficients were less than 20%. The colonization of SE in organs increases the enzyme activities of AST and ALT in blood.

**Conclusions:**

SE in feed could invade the oviduct and infect the forming eggs. It significantly increased the concentration of ALT and AST in blood. However,SE infection was hard to be observed from the appearances of layer and egg. It might be a dangerous risk to human health.

## Background

In the late 1970s, poultry flocks infected by Salmonella enterica serovar Enteritidis (SE), which was asymptomatic, was reported [1]. By the mid 1980s, SE spread rapidly throughout the United Kingdom, the United States, South America, and other countries [2,3]. Bäumler et al. and Rabsch et al., attributed this to successful campaigns to eradicate the Salmonella serovars Gallinarum [4,5]. Which had became a major cause of foodborn illness. Contaminated chicken eggs are an importance agent for the transmission of SE to humans [6-9], especially from consuming eating raw shell eggs and inadequately cooked eggs [10,11]. The risk of human infections following consumption of SE-contaminated eggs depends on the bacterial number present [12]. An egg-associated SE outbreaked in US in 2010, led to a nationwide recall of more than 500 millions eggs with nearly 2,752 reported illnesses [11]. Egg contamination issues affected not only public health but also the food industry itself, causing costly recalls and damage to the reputations of brand-name products [13].

SE is considered as the only bacterium that routinely causes human infection through intact chicken eggs [9,14]. Although an egg has its own protection mechanisms including both physical and chemical barriers, with bactericidal factors such as lysozyme, ovotransferrin, nuclease and β-defensin-11existed in egg albumen that can kill most bacteria. However, SE has a series of unique mechanisms to survive and multiply in the internal egg contents [11].

Two possible routes of egg contamination by SE, include the colonized gut where contaminated feces can penetrate the eggshell during or after ovipositing (horizontal transmission). Another involves infected reproductive organs that contaminate egg contents directly before ovipositing (vertical transmission or transovarian transmission). Several lines of evidence support the view that egg are mainly contaminated with SE through the vertical transmission. That is SE could escape the host defense and colonize in the reproductive organs including the ovaries and oviduct and thus contaminate the yolk and albumen directly before oviposition [15-17]. Because SE could colonize all sites in the hen reproductive tract, contamination of any part of the egg is possible and environmental hygiene, bacteria vectors such as birds, flies, rodent, and beetles along with feed contamination can be major causes of SE colonization in hens, and thus to eggs.

Although various control measures had been adopted throughout the food production chain, the microbiological testing of eggs during production and processing remain an important role in preventing food-borne infection [18]. However, the traditional cultural isolation method for detecting SE requires up to 5 d to 7 d and thus delays diagnosis. Methodology based on PCR is simple, rapid, and specific with high sensitivity. Although it is competent for fast identification and detecting pathogenic microorganisms, including SE, PCR method egg is dependent on specific genes and primers. SE and some other serovars, such as Dublin and Pullorum, the two closest relatives of SE, shared remarkable similarities in both linear organization and sequence of the genomes, with sequence homologies of the conserved regions ranging from 96% to 99% [19,20]. Moreover SE still could differ significantly from another serovar in several characteristics including specific genes [21]. For example, a unique 60-kb virulence plasmid of SE possesses the Prot6E gene whose encoding has a unique surface fimbriae specific to SE [21,22]. The fimbriae played a role in the interaction with egg albumen components. Malorny et al. proved the specificity of Prot6E with 54 serovars [23].

In a previous study, we had tested the specificity of Prot6E in poultry and egg samples, and developed a fast and sensitive PCR method for specific detection of SE. In the present study, we focused on the colonizing role of SE in hen organs and forming eggs (internal eggs before they were laid), by feeding the egg-laying hens with the feed contaminated by SE.

## Methods

### Salmonella strain

*Salmonella enterica* ssp. *enterica* serovar Enteritidis (*S.* Enteritidis) obtained from China Institute of Veterinary Drugs Control.

### Birds

One hundred SE negative hens were randomly divided into 2 groups, 40 to control group, 60 to experimental group and transferred to two isolation rooms, where they were housed in individual standard wire mesh cages. Blood was obtained from the brachial vein of 20-wk-age White Leghorns hens came from China Agricultural University (CAU), and assayed for SE by the whole-blood plate agglutinate test [24]. Animals were handled in accordance with the principles and procedures outlined by the China Agricultural University’s Animal Care and Use Committee.

### Feed

Feed purchased from China Chia Tai Feed Co., Ltd. Consist of cereal, bran, soybean meals, rapeseed meal, fish meal, calcium hydrophosphate, vitamin A, D, E, K, B, minor element including Cu, Fe, Mn, Zn. The percentage of the main feed ingredients as follows, moisture 13.0%, crude protein 16.5%, crude fibre 5.0%, crude ash 13.0%, calcium 3.60%, gross phosphorus 0.65%, salt 0.34%, methionine and cystine 0.68%.

Before and after the experiment, six 15 g samples of feed were randomly taken from every bag, and enriched successively with cultured bacteria Buffer Peptone Water (BPW) and Selenite Cystine Broth (SC). Using PCR extract bacterial DNA were used to detect whether the feed was contaminated by SE. The results showed that the feed without artificial infection did not contain SE, which would ensure the real SE infection level for the experiment.

### Primers design, DNA preparation and DNA amplification

The sequences of the primer pairs used for PCR detecting were designed according to the SE special Prot6E nucleotide sequence (NO. U66901), the production size was 175 bp. The sequences of the primer pairs were as follows: Prot6E-F: 5′-ACAGGGGCACAATAACCGTA-3′ and Prot6E-R: 5′-TGCATCCCTGTCACAACATT-3′.

Every sample was put in a culture plate and homogenized with BPW according the rate 1:9, and incubated at 37°C for 24 h to allow for bacterial growth. After incubation, injecting 1 ml of the preenrichment broth into 9 mL of SC and incubated at 37°C for 24 h. Then, 1 ml of the selective enriched sample was transferred to a microcentrifuge tube with a capacity of 2 mL. The cell suspension was centrifuged for 5 min at 12,000 rpm. The supernatant was discarded, and then the pellet was washed twice by ddH_2_O, and suspended in 200 μL ddH_2_O. The micro-centrifuge tube was boiled for 15 min and immediately chilled on ice for 2 min. Then the tube was centrifuged for 15 min at 12,000 rpm. The supernatant was then transferred to a new micro-centrifuge tube and used as the template DNA in the PCR.

The amplification reaction was performed in a total volume of 15 μL as follows: 1.5 μL of 10 × PCR buffer, 0.8 μL of dNTP, 0.15 μL of primer Prot6E-F and 0.15 μL of primer Prot6E-R, 1.0 μL of DNA, 0.3 μL of Ex Taq enzyme, 11.1 μL of ddH2O. The reaction mixture was run online at 94°C for 5 min, followed by 35 cycles at 94°C for 40 s, 60°C for 40 s, 72°C for 50 s, with an extension phase of 1 cycle at 72°C for 10 min. Samples were fractionated by 1.5% agarose gel electrophoresis and visualized by ethidium bromide staining.

### Supplementary SE to hens

Two wks pretest were set to record the laying rate (LR) and egg weight (EW) to ensure they were similar on the first day of experiment, the hens in experimental group were fed with 30 g feed contaminated 9.8 × 10^8^ CFU SE, the control group hens were fed the feed without SE. Then two groups were fed the feed without SE and raised in the same situation.

### Detecting SE in internal organs, reproductive tract and forming eggs

On the 8 d and 16 d after supplementary SE, 10 hens from the control group and 30 hens from the experimental group were euthanized by intravenous injection of T61, and anatomized respectively. Cecal contents, heart, liver, spleen, follicle, ovary, oviduct including infundibulum, magnum, isthmus, uterus, vagina, and forming eggs in the oviduct were collected, and the colonizing of SE evaluated by PCR. The SE on eggshell were sampled and processed within 24 h. Results were showed in Table 1.

**Table 1:** Colonization rate (%) of SE in tissues and forming eggs

	**8 d**		**16 d**	
**Location**	**Controls**	**Experiments**	**Controls**	**Experiments**
**N**	**10**	**30**	**10**	**30**
Caecum content	0	55.17	0	33.33
Heart	0	31.03	0	33.33
Liver	0	34.48	0	30.00
Spleen	0	44.83	0	36.67
Follicle	0	13.79	0	23.33
Ovary	0	20.69	0	33.33
Infundibulum	0	24.14	0	20.00
Magnum	0	24.14	0	26.67
Isthmus	0	24.14	0	23.33
Uterus	0	31.03	0	26.67
Vagina	0	17.24	0	23.33
Egg yolk	0	18.81	0	14.81
Egg albumen	0	22.73	0	22.22
Eggshell membrane	0	40.91	0	37.50
Eggshell	0	80.00	0	62.50

### Measured blood biochemical indices, production performance and egg qualities

Blood from the brachial vein were collected in the 8 d and 16 d after supplementary SE, and blood biochemical indices, including alanine aminotransferase (ALT), aspartic aminotransferase (AST), lactic dehydrogenase (LDH), glucose (GLU) and total protein (TP) determinal.

Fourteen d before supplementary SE (pretest) and 16 d after supplementary SE, laying rate and egg weight were recorded daily. Eggshell strength (ESS), eggshell thickness (EST) and eggshell ratio (ESR) were measured daily for 16 d after supplementary SE. Data were on the individual bird basis.

### Statistical analysis

The units for analysis were the average values for each hen. All statistical analyses were performed using SAS procedures, *t*-test (SAS Institute, Cary, NC, USA).

## Results

### The colonization of SE in hens and forming eggs

After hen was infected by SE, the colonization rate of SE was highest in caecum contents, followed by visceral organs, and lowest in reproductive tract (Table 1). Among the visceral organs, the positive rate was highest in spleen, and the spleen was tumefaction obviously, its weight was significhantly heavier than the control group (*P* < 0.05) (Table 2).

**Table 2:** Effect of SE infection on the chicken’s spleen weight

**Date**	**Spleen weight, g**	
	**Controls, N = 10**	**Experiments, N = 30**
8 d	1.24 ± 0.32^b^	1.52 ± 0.33^a^
16 d	1.22 ± 0.32^b^	1.63 ± 0.62^a^

^**a,b**^Means in same row with a day lacking a common superscript differ significantly *(P* < 0.05).

In the reproductive tract, the colonization rate of SE was highest in uterus, moderately high in isthmus and magnum, and lowest in follicles. For the forming eggs, the detection rate of SE was highest on eggshell, followed by eggshell membrane, albumen, and lowest in and yolk. This pattern was consistent with the colonize tendency in the corresponding site of the reproductive tract.

### Blood biochemical indices

The blood analysis suggested that the AST and ALT of experimental group was significantly higher than that of control group *(P* < 0.05) (Table 3). The GLU, LDH and TP of experimental group were similar to that of controls *(P* > 0.05).

**Table 3:** Effect of SE infection on the blood biochemical indices

	**8 d**		**16 d**	
**Items**	**Control**	**Experiment**	**Control**	**Experiment**
AST, U/L	211.84 ± 31.63^a^	236.22 ± 33.37^b^	269.04 ± 39.51^a^	308.91 ± 33.93^b^
ALT, U/L	24.55 ± 7.55^a^	32.19 ± 10.31^b^	22.70 ± 7.05^a^	32.37 ± 13.73^b^
GLU, mmol/L	10.83 ± 0.77	10.91 ± 0.55	10.28 ± 0.69	10.46 ± 0.98
LDH, U/L	174.80 ± 36.99	160.22 ± 63.07	211.27 ± 62.73	191.10 ± 55.63
TP, g/L	60.38 ± 7.23	60.79 ± 7.65	61.58 ± 5.15	61.78 ± 7.96

^**a,b**^Means in same row for each age lacking a common superscript differ significantly *(P* < 0.05).

### Laying rate and egg quality

The laying rates and egg weight of controls and experiments were similar during the 14 d pretest, and then the SE infection didn’t influence two traits *(P* > 0.05) (Table 4).

**Table 4:** Mean and standard deviation for eggshell quality

**Trait**	**Controls**	**Experiments**
Laying rate, %	82.44 ± 4.45	82.19 ± 4.12
Egg weight, g	41.79 ± 3.47	42.54 ± 3.69
Egg thickness, mm	0.314 ± 0.024	0.308 ± 0.023
Eggshell strength, kg/cm^2^	3.325 ± 0.570	3.174 ± 0.561
Eggshell weight, g	4.76 ± 0.37	4.72 ± 0.45
Eggshell ratio, %	11.53 ± 0.84^a^	11.15 ± 0.67^b^

^**a,b**^Means in same row with lacking a common superscript differ significantly (*P* < 0.05).

Egg thickness, eggshell strength, eggshell weight and eggshell ratio of experimental group were all lower than that of the controls *(P* < 0.05) (Table 4).

## Discussion

It is well known that traditional cultural isolation of SE is labrious and time-consuming and PCR methods were introduced in the 1990 as simple and faster methods to detect pathogenic microorganisms. The Prot6E gene had been reported as specific for SE, it located on a 60-kb virulence plasmid which was important in the pathogenicity of SE strains [25]. In our previous study, a pair of special primers tested the specificity of Prot6E gene, and showed that the PCR assay was selectivity, accuracy, and applicable. This PCR assay could be used to identify SE or to detect SE directly from poultry tissues or eggs [26].

After hens consumed the feed contaminated by SE, SE entered into esophagus, invaded and passed through intestinal epithelial cell, entered the mesenteric lymph nodes, then spread around the body carried by macrophages. SE could infect and colonize in the spleen, liver, heart, bone marrow and other organs and organizations [16,27-29]. The detection of SE was highest in cecal contents, followed by visceral organs, and lowest in reproductive tract, was consistent with previous researches [30,31]. Among internal organs, the colonization rate in spleen was highest, the reason might be the spleen played a role in filtering blood, and SE spread within the host carried by the macrophages. When SE reached to spleen, huge amounts of macrophages with SE could invade into the spleen tissue. Because of its capability to infiltrate, survive and replicate in the immune cells [31], SE could stimulate the macrophages and lymphocyte to proliferate, resulting in the spleen congestion and tumefaction. The colonization rate of SE in heart and liver was same high, and damage their functions, which could be reflected by the significantly rise of the concentration of ALT and AST. ALT mainly existed in liver cells and is closely related to liver cell activity. AST mainly existed in heart, followed by liver. When the heart and liver are damaged badly, the level of ALT and AST could increase significantly [32].

SE could invade and colonize in each part of oviduct. Starting from the ovary, the infundibulum captured the ovulatory follicles, the magnum produced the albumen, the isthmus deposited the eggshell membranes, the uterus forms the eggshell and the vagina was involved in oviposition. Colonizing of SE in the oviduct might be infect the yolk, albumen, eggshell membrane and eggshell accompanying the egg formation [31,33,34]. The infection would not end the formation process of eggs, while SE would not be killed by the antibacterial substances secreted by oviduct. This implied that SE has internal factors or induces factors to counteract the chicken’s antibacterial system, and shown a unique live mechanism in the egg formation process.

SE passed serial passage from the liver to spleen, and does not affect its ability to induce egg contamination. However, the repeated *in vivo* passages in the reproductive tissues could enhance the ability of SE to cause egg contamination [35]. The results of our study showed that after supplementary SE to hens, the colonization rate of SE in different sites of the reproductive tissues was changed. The colonization rates in follicle, ovary, infundibulum were relatively low, while the colonization rate in uterus was comparatively high. This could lead to the significant differences in contamination rates of SE in different components of the egg.

In this study, The detection of SE in eggshell was highest, moderately high in eggshell membrane and albumen, and the detection in yolk was lowest, which was basically consistent with the colonization rate in the corresponding segment of oviduct. The colonization rate in uterus was obviously higher than other segments, consisting with the higher SE comtamination on the eggshell. However, the colonization rates were not same level. For example, the detection of SE of eggshell was higher than that in uterus. There were some reasons might explain this phenomenon. The SE on the eggshell came not only from colonization in uterus, but also may be influenced by colonization in vagina which could ascend to uterus [36]. The detection of SE in eggshell membrane was higher than that of isthmus. It reflected that eggshell membrane contamination does not only caused by the directly contaminated by SE colonization in isthmus, but also SE in albumen might adhere to eggshell membrane, and SE on eggshell might penetrate onto the membrane. The detection of SE in egg albumen can be lower than that of magnum. The pH of the egg albumen was alkaline [37], and the albumen contains antibacterial factors including nuclease, lysozyme, ovotransferrin and β-Defensin-11 [38-41], they could forbid or kill SE [9]. The detection rate in egg yolk was lower than that of ovary. Other studies have suggested that SE had the ability to penetrate into the yolk, when it combine with type 1 fimbriae on vitelline membrane [33,42]. However, the vitelline membrane also had the same antibacterial factors as in albumen [43].

Simulation experiments had shown that various bacterial species could cause internal contamination of egg [44-46]. Under normal production and storage conditions, this was a rare event. When eggs had been damaged or cracked, they could be contaminated by bacteria and deteriorated quickly and could be easily identified. In our study, after supplementary SE to hens, SE colonized in the oviduct and contaminated the forming eggs. The large number of SE colonized in uterus might influence eggshell qualities, although the contamination of the egg contents didn’t lead to an abortive egg formation. The egg weight, eggshell strength, eggshell thickness and eggshell weight didn’t change by SE contamination. One study observed the growth of SE in eggs did not lead to phenotypic changes in the color, smell and consistency of the egg contents [47]. However, SE kept growing in albumen after eggs output, and it also might penetrate into yolk [48-50].

## Conclusions

In the present study, we designed a specific PCR to test the SE contamination in feed, organ and egg. The chicken infected with SE through feed had no significant changes on production performance and egg qualities. So the laid eggs infected by SE were hard to be discerned by the consumer and became seriously risk to human health.

## Competing interests

The authors declare that they have no competing interests.

## Authors’ contributions

FS carried out the experiments and drafted the manuscript. JZ undertook the part of research and wrote the manuscript for publication. GX advised FS in designing and performing the experiment. DZ and NY provided a lot of help with the project. All authors have read and approved the final manuscript.
